# Damage-free highly efficient plasma-assisted polishing of a 20-mm square large mosaic single crystal diamond substrate

**DOI:** 10.1038/s41598-020-76430-6

**Published:** 2020-11-10

**Authors:** Nian Liu, Kohki Sugawara, Naoya Yoshitaka, Hideaki Yamada, Daisuke Takeuchi, Yuko Akabane, Kenichi Fujino, Kentaro Kawai, Kenta Arima, Kazuya Yamamura

**Affiliations:** 1 Department of Precision Engineering, Graduate School of Engineering, Osaka University, 2-1 Yamadaoka, Suita, Osaka 565-0871 Japan; 2 TDC Corporation, 24-15 Chojamae, Aza, Iidoi, Rifucho, Miyagi District, Miyagi 981-0113 Japan; 3 Diamond Material Team, Advanced Power Electronics Research Center, National Institute of Advanced Industrial Science and Technology (AIST), 1-8-31 Midoriga-oka, Ikeda, Osaka 563-8577 Japan

**Keywords:** Mechanical engineering, Semiconductors

## Abstract

Plasma-assisted polishing (PAP) as a damage-free and highly efficient polishing technique has been widely applied to difficult-to-machine wide-gap semiconductor materials such as 4H-SiC (0001) and GaN (0001). In this study, a 20-mm square large mosaic single crystal diamond (SCD) substrate synthesized by microwave plasma chemical vapor deposition (CVD) was polished by PAP. Argon-based plasma containing oxygen was used in PAP to modify the surface of quartz glass polishing plate, and a high material removal rate (MRR) of 13.3 μm/h was obtained. The flatness of SCD polished by PAP measured by an interferometer was 0.5 μm. The surface roughness measured by both scanning white light interferometer (SWLI) (84-μm square) and atomic force microscope (AFM) (5-μm square) was less than 0.5 nm *S*q. The micro-Raman spectroscopy measurement results of mosaic SCD substrate processed by PAP showed that residual stress and non-diamond components on the surface after PAP processing were below the detection limit.

## Introduction

Single crystal diamond (SCD) has many excellent electronic, chemical, and mechanical properties, such as a large band gap, high electron mobility, high breakdown electric field, low thermal coefficient of expansion, and the highest thermal conductivity as known. Therefore, it has been widely considered as the most ideal material for the fabrication of electronic and optical devices that can withstand extreme conditions such as high input power and frequency, low or high temperature, and corrosive environments^1^. As the significant advancement in material science, it is possible to economically synthesize high-quality and large-sized SCD substrate by microwave plasma chemical vapor deposition (CVD). Currently, the National Institute of Advanced Industrial Science and Technology (AIST) in Japan has successfully fabricated inch-sized mosaic SCD wafers by the clone method^2^. To take full advantage of the excellent properties of SCD substrate in the abovementioned application, an atomically smooth surface without scratches, subsurface damage, and non-diamond components is essential^3^. However, polishing of diamond is very difficult because it is the hardest of all materials and is also chemically inert. At present, scaife polishing using a metal-bonded diamond wheel or a cast iron surface plate in which diamond powder is embedded is used to polish diamond. Polishing with hard abrasive grains, such as diamond, forms a serious damage layer on the surface or subsurface region of the polished diamond substrate^4^.

Various polishing methods that combine mechanical, chemical, and thermal actions have been proposed for highly efficient and damage-free polishing of diamond substrates. Ultraviolet (UV)-irradiated polishing was proposed and applied to a 3 mm × 3 mm × 1 mm CVD diamond. The material removal rate (MRR) of the SCD (100) substrate was promoted by the photoexcitation effect of UV irradiation. As a result, an MRR of 0.5 μm/h, 1.7 times as high as that without UV irradiation, was achieved^5^. Kubota et al*.* demonstrated a polishing technique using a chemical reaction with an H_2_O_2_ solution. The diamond substrates used as samples were fabricated by high-pressure high-temperature (HPHT) method, and the size was 3 mm × 3 mm × 1.5 mm. The hydroxyl radicals generated by the decomposition of H_2_O_2_ were considered to be a factor that improved the MRR of the SCD substrate. An MRR of 216.7 nm/h, 3 times as high as that without feeding of H_2_O_2_ solution, was obtained^6^. Dynamic friction polishing (DFP) using a metal disk was proposed and applied to polish an φ12.7 mm × 4 mm polycrystalline diamond (PCD) specimen. In this method, it is assumed that the non-diamond component, which is converted by the reaction between the diamond surface and the catalytic metal at high temperature caused by friction, is mechanically or chemically removed. A large amount of non-diamond components is detected by Raman spectroscopy on the surface of diamond polished by DFP. Furthermore, the high polishing pressure and high sliding speed tend to cause the diamond substrate to crack^7^.

Plasma-assisted polishing (PAP) was proposed by our research group as a novel technique for efficiently polishing diamond without introducing any damage. When an SCD (100) substrate with an area of 93 mm^2^ was polished by PAP using an argon plasma containing water vapor and a polishing plate made of quartz glass, a polishing rate of 2.1 μm/h was obtained. When the SCD substrate was polished by PAP using a sapphire polishing plate, a surface roughness of 0.13 nm *S*q was obtained. Furthermore, micro-Raman spectroscopy showed that the crystallinity of the SCD surface before and after PAP did not change; therefore, polishing of diamond by PAP was found to be damage-free^8^. In the present study, we describe the PAP polishing characteristics of 20-mm square mosaic SCD substrate made by the clone method.

## Results

### Morphology of the mosaic SCD (100) substrate surface before PAP

**Figure 1:**
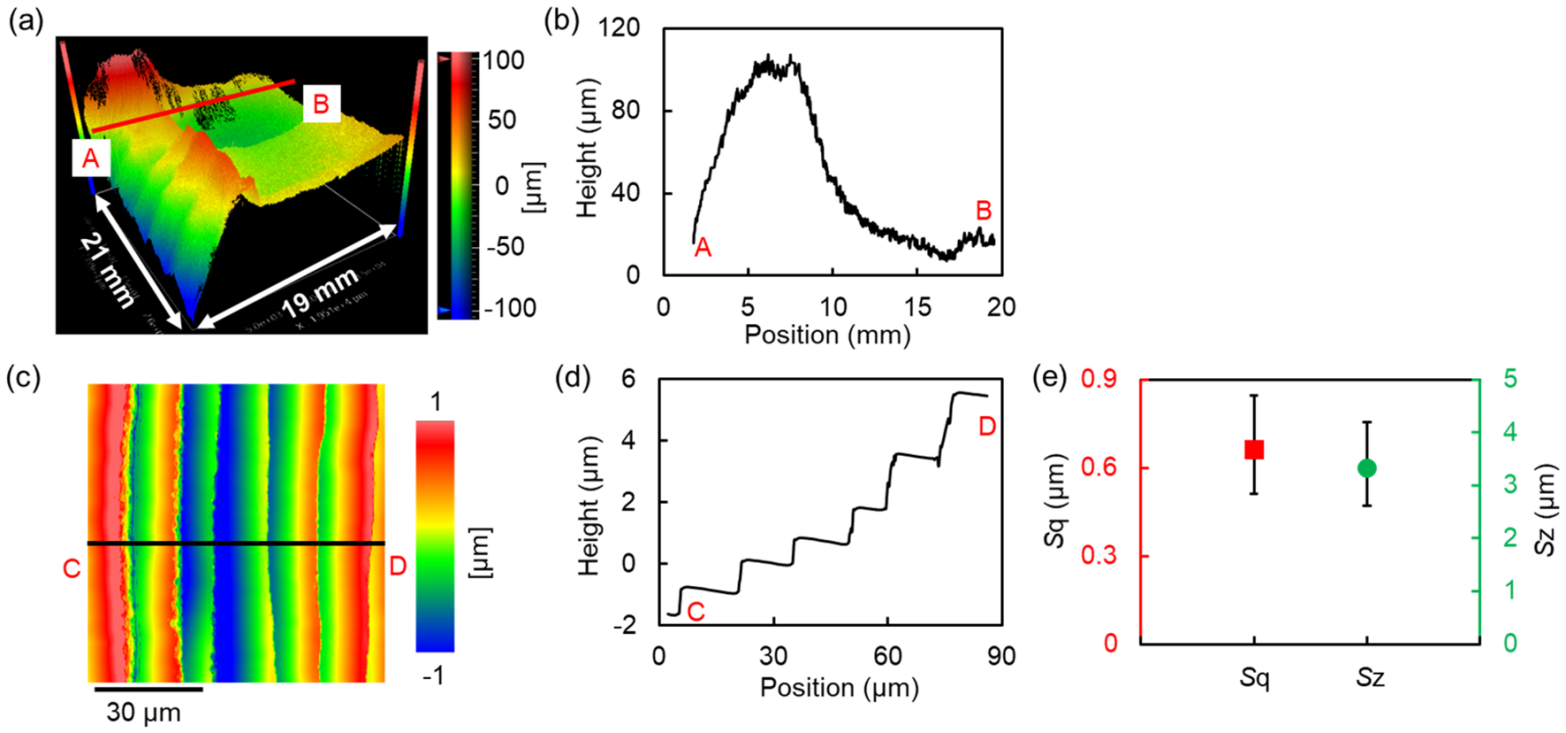
Surface morphology of the 20-mm square mosaic SCD (100) substrate before PAP. (**a**) Three-dimensional SWLI image of the entire mosaic SCD substrate before PAP obtained by the stitching application; (**b**) cross-sectional profile of A–B; (**c**) SWLI image of one local area on the mosaic SCD substrate before PAP, *S*q: 0.569 μm, *S*z: 2.982 μm; (**d**) cross-sectional profile of C–D; (**e**) the average and distribution of *S*q*, S*z value of seven different local areas on the mosaic SCD substrate before PAP obtained by SWLI (84-μm square), the error bars here indicate the distribution of the values of the measured *S*q roughness and *S*z roughness between the minimum and maximum local areas.

In order to fully characterize the surface morphology of the 20-mm square mosaic SCD substrate, a scanning white light interferometer (SWLI) microscopy with a stitching application was used. Figure 1a shows the three-dimensional SWLI image of the 20-mm square mosaic SCD substrate before PAP. As shown in Fig. 1b, a large waviness with a height of approximately 100 μm formed during microwave plasma CVD growth was observed. To evaluate the surface roughness of the mosaic SCD substrate, seven local areas on the entire substrate were measured by SWLI. An SWLI image of one local area and its cross-sectional profile are shown in Fig. 1c, d and Fig. 1e shows the average and distribution of the measured *S*q roughness and *S*z roughness at seven measured local areas. As shown in Fig. 1d, a step bunching structure with a terrace width of approximately 10 μm, and a step height of 1–2 μm was formed on the surface of the mosaic SCD substrate made by the microwave plasma CVD growth, and the average *S*q roughness was 0.66 μm. During the microwave plasma CVD growth process of the SCD substrate, nitrogen was introduced to increase the deposition rate. The addition of nitrogen benefited to create a macro smooth surface by avoiding the growth of hillocks. However, it created a micro rough surface by generating step-bunching structure^9^, as shown in Fig. 1c–e.

### The MRR of the mosaic SCD (100) substrate during PAP

**Figure 2:**
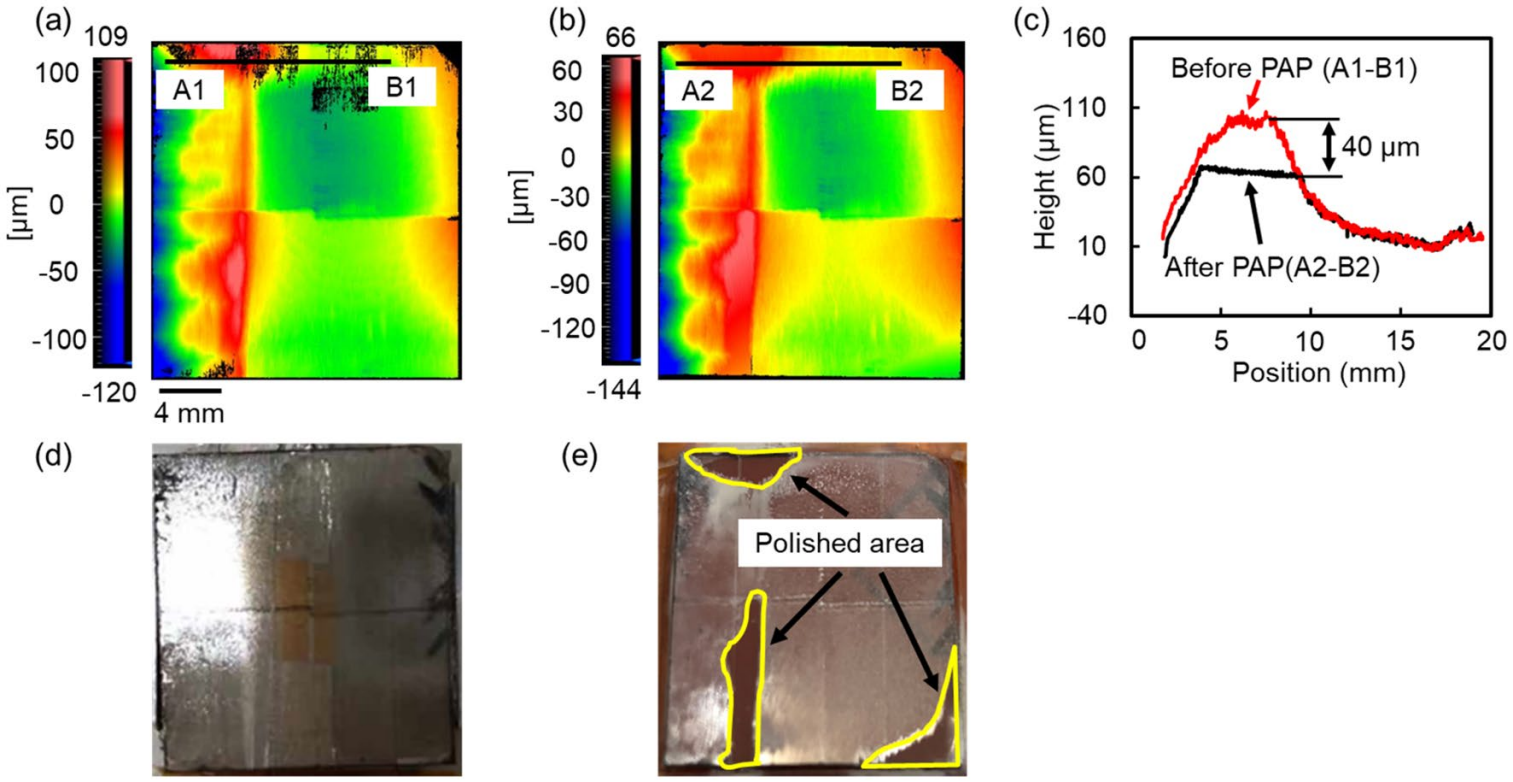
Changes in the surface morphology of the mosaic SCD (100) substrate before and after PAP. (**a**) SWLI image of the CVD-grown mosaic SCD substrate before PAP; (**b**) SWLI image of the mosaic SCD substrate after PAP for 3 h; (**c**) cross-sectional profile of the same point on the mosaic SCD substrate before and after PAP for 3 h; (**d**) photo of the mosaic SCD substrate before PAP; (**e**) photo of the mosaic SCD substrate after PAP for 3 h.

Figure 2a,b show the SWLI images of the mosaic SCD substrate before and after PAP, respectively. Figure 2c shows the cross-sectional profile before and after PAP at the same point on the mosaic SCD substrate, which indicates that the polishing depth by PAP for 3 h was 40 μm. Therefore, the MRR for the 20-mm square mosaic SCD substrate during the initial 3 h of PAP was 13.3 μm/h. The photographs in Fig. 2d,e show that polishing by PAP proceeded from the highest point of the waviness on the mosaic SCD substrate. Because the polished area after 3 h of PAP calculated from the SWLI image was 42 mm^2^, the polishing pressure was calculated to be 401 kPa assuming that the average contact area in the first 3 h was 21 mm^2^.

### Morphology of the mosaic SCD (100) substrate surface after PAP

**Figure 3:**
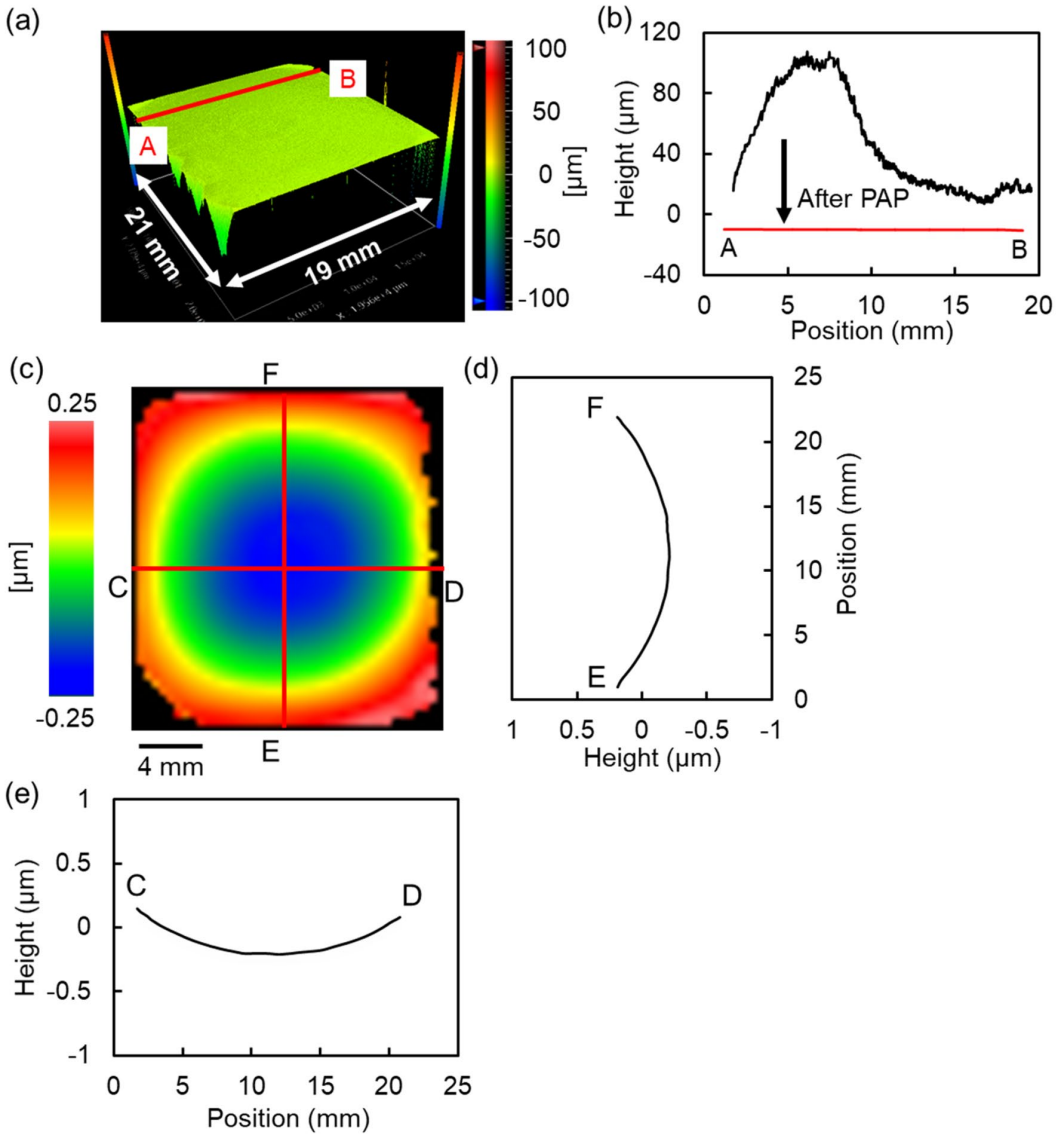
Morphology of the 20-mm square mosaic SCD (100) substrate after PAP. (**a**) Three-dimensional SWLI image of the entire mosaic SCD substrate after PAP obtained by the stitching application; (**b**) cross-sectional profiles of the same point on the mosaic SCD substrate before and after PAP; (**c**) flatness of the PAP-processed mosaic SCD substrate measured by a laser interferometer; (**d**) cross-sectional profile of E–F; (**e**) cross-sectional profile of C–D.

Figure 3a,b show the shape of the PAP-processed mosaic SCD substrate obtained by the stitching measurement using SWLI and A-B cross-sectional profiles at the same point on the mosaic substrate before and after PAP, respectively. These results show that the large waviness (height ≥ 100 μm), which was on the CVD-grown mosaic SCD substrate before polishing was completely removed and planarized by PAP. Figure 3c–e show the flatness measured by a laser interferometer and its cross-sectional profiles along two perpendicular directions, respectively. A flatness of 0.5 μm or less was obtained by applying PAP to the polishing of the CVD-grown mosaic SCD substrate.

**Figure 4:**
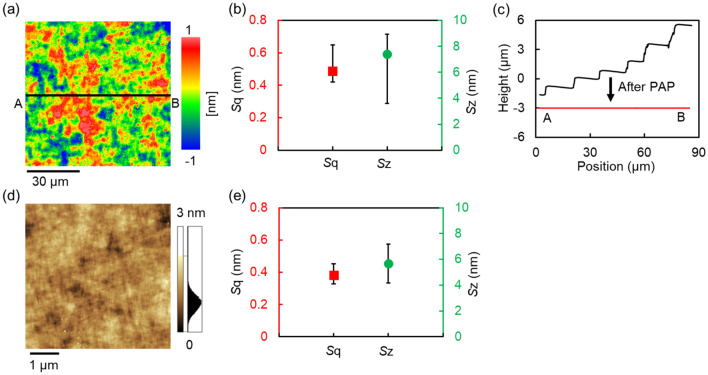
Surface roughness of the 20-mm square mosaic SCD (100) substrate after PAP. (**a**) SWLI image of one local area on the mosaic SCD substrate after PAP, *S*q: 0.458 nm, *S*z: 8.884 nm; (**b**) the average and distribution of *S*q*, S*z value of seven different local areas on the mosaic SCD substrate after PAP obtained by SWLI (84-μm square); (**c**) cross-sectional profiles of the same point on the mosaic SCD substrate before and after PAP; (**d**) AFM image of one local area on the mosaic SCD substrate after PAP, *S*q: 0.361 nm, *S*z: 5.169 nm; (**e**) the average and distribution of *S*q*, S*z value of seven different local areas on the mosaic SCD substrate after PAP obtained by AFM (5-μm square).

To evaluate the roughness component on the polished surface, the mosaic SCD substrate after PAP was measured by SWLI and atomic force microscope (AFM). Figure 4a–c show a surface morphology of one local area, the average and distribution of the measured *S*q roughness and *S*z roughness of seven local areas on the mosaic SCD substrate after PAP, and cross-sectional profiles before and after PAP, measured by SWLI, respectively. Figure 4a,b indicate that an *S*q roughness of approximately 0.5 nm was uniformly obtained by PAP on the entire surface of the mosaic SCD substrate. Figure 4c indicates that the step-bunching structure with a step height of 1–2 μm was completely removed by PAP. Figure 4d,e show a surface morphology of one local area, the average and distribution of the measured *S*q roughness and *S*z roughness of seven local areas on the mosaic SCD substrate after PAP, measured by AFM, respectively. An *S*q roughness of approximately 0.4 nm was obtained on the entire surface of the mosaic SCD substrate in the high spatial frequency region. Moreover, the results of SWLI and AFM indicate that there were no scratches on the mosaic SCD substrate surface after PAP. When an SCD (100) is polished by a mechanical method, such as scaife, the polishing rate in the <100> direction is much higher than that in the <110> direction. Thus, streak-like structures are easily formed on the surface^10,11^. On the other hand, because the surface texture depending on the crystal direction is not observed on the SCD (100) surface polished by PAP, it is presumed that not only mechanical action exists in the PAP of SCD^8^.

### Crystallinity of the mosaic SCD (100) substrate surface after PAP

**Figure 5:**
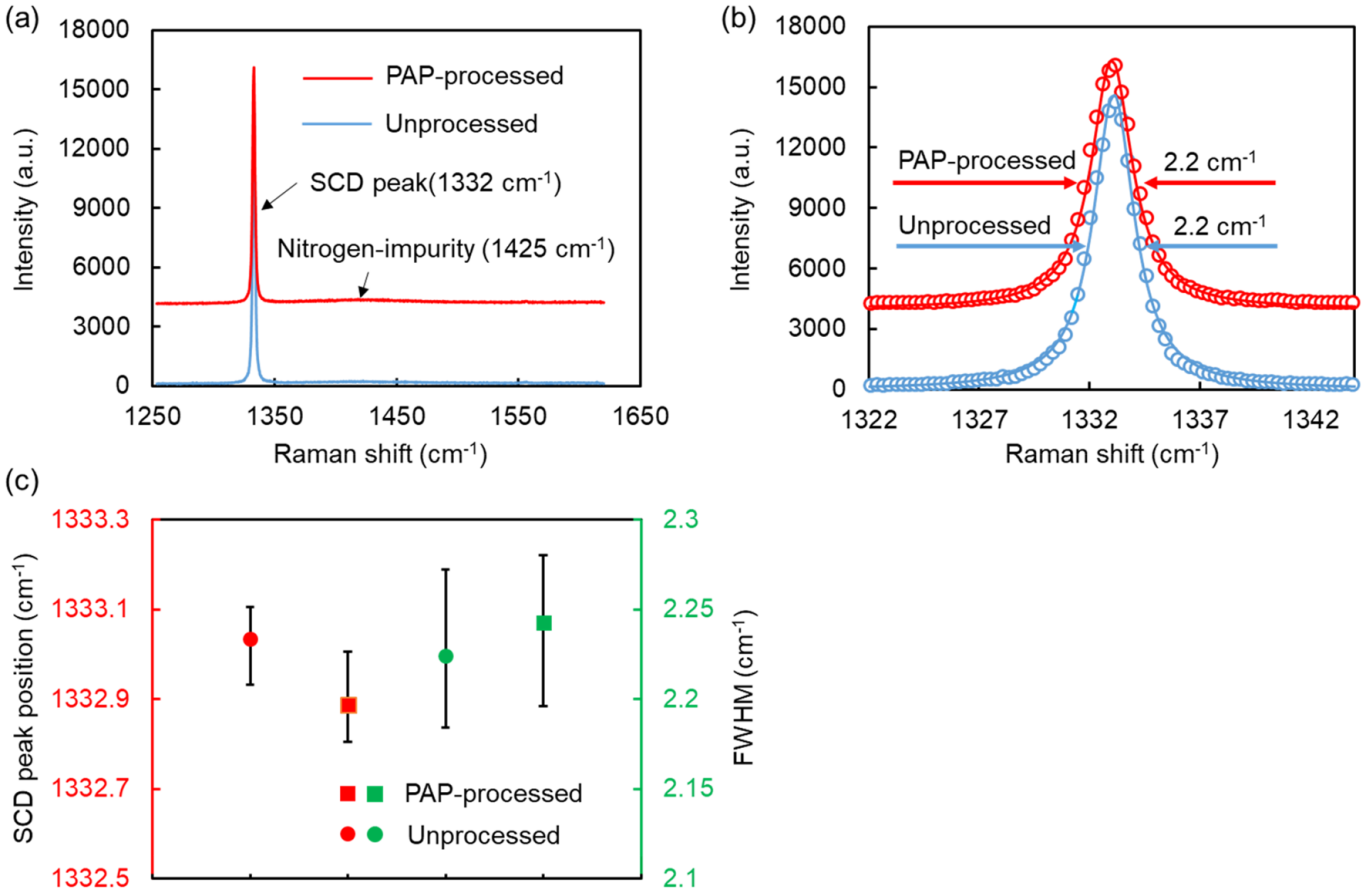
Micro-Raman spectra of different points on the unprocessed and PAP-processed areas of the 20-mm square mosaic SCD (100) substrate. (**a**) Micro-Raman spectra of one group of points; (**b**) magnified diamond Raman lines shown in (**a**); (**c**) the average and distribution of the peak position and FWHM of the diamond Raman lines for the different measured points, the error bars here indicate the distribution of the values of the peak position and FWHM of the diamond Raman lines between the minimum and maximum points. After the mosaic SCD (100) substrate was polished by PAP, a narrow area in the left edge of the substrate was not polished as shown in Fig. 3a, which was named as “unprocessed”. On the contrary, other polished flat area was named as “PAP-processed”.

To evaluate the crystallinity of the SCD surface polished by PAP, seven points on the unprocessed and PAP-processed areas, respectively, of the 20-mm square mosaic SCD (100) substrate were measured by confocal Raman microscopy. The wavelength and spot size of the excitation laser beam in Raman spectroscopy were 532 nm and 300 nm, respectively. Figure 5a shows micro-Raman spectra of one group of points on the unprocessed and PAP-processed areas of the mosaic SCD substrate. The broad band at 1425 cm^−1^ originates from the nitrogen addition to increase the deposition rate in the SCD deposition using microwave plasma CVD^9^. No Raman band originating from the G band of graphite (1580–1600 cm^−1^)^12^ was observed. Figure 5b shows the magnified diamond Raman lines shown in Fig. 5a, which was fitted with a Lorentzian function due to the infinite periodicity of crystal lattice in SCD substrate^13^. Figure 5c shows the average and distribution of the peak position and full width at half maximum (FWHM) of the diamond Raman lines for the different measured points. The average peak position of the diamond Raman lines measured on the unprocessed and PAP-processed areas were 1333.0 cm^−1^ and 1332.9 cm^−1^, respectively. The average FWHM of the diamond Raman lines on the both unprocessed and PAP-processed areas were the same, 2.2 cm^−1^. In the case of a perfect diamond lattice, the peak position of the diamond Raman line is 1332.5 cm^−1^, and the FWHM of the line is approximately 1.5 cm^−1^. The slight shift and broadening of diamond Raman line position and FWHM of the line are considered to be caused by the imperfection of the crystallinity from several reasons, for instance, impurity such as nitrogen in the mosaic SCD substrate^14^. When dynamic friction polishing (DFP), which has mechanical, thermal, and chemical effects as removal phenomena, was applied to polish SCD, the FWHM of diamond Raman line broadened from 2.7 to 5.49–9.75 cm^−1^, indicating that subsurface damage (SSD) was formed on the surface of SCD^15^. When mechanical ultrasonic polishing using a water-based slurry with diamond grit was applied to polish the polycrystalline diamond (PCD), the FWHM of diamond Raman line broadened from 3.8 ± 0.7 to 5.3 ± 0.4 cm^−1^. Also, the broadening of FWHM of the diamond Raman line was observed in a mechanical scaife polishing using diamond grits. In addition, the Raman bands at 1590 cm^−1^ and 1606 cm^−1^ attributed to the G band of *sp*^2^ (1500–1630 cm^−1^)^12^, were observed respectively at these two mechanical polishing methods, indicating that diamond structure was destroyed due to excessive mechanical stress^16,17^.Therefore, PAP is an ideal polishing technique for SCD because it does not bring about a disordered or amorphous carbon phase that causes the broadening of FWHM of the diamond Raman line while has an MRR of 10 μm/h or higher.

## Discussion

A previous study has reported that polishing of diamond with a soft SiO_2_ wheel results in a much faster MRR than mechanical polishing with a metal-bonded diamond wheel, due to the synergistic effect of chemical and mechanical action^11^. It has been reported in our previous research that the polishing rate of SCD was increased by 20 times by irradiating the quartz glass polishing plate with plasma. Therefore, it is assumed that in the PAP of SCD, the chemical removal action is dominant rather than mechanical action^8^. The SiO_2_ weakening model proposed by Peguiron^18^ can be used to explain the chemical removal mechanism in the PAP of SCD. At first, silica can chemically weaken the C–C bonds when sliding against a diamond surface. Then, Si–C and O–C bonds with the strength that is higher than the weakened C–C bonds can be generated at the diamond/silica interface. As a result, the C atoms on the diamond can be extracted by the relative motion between the mosaic SCD substrate and the polishing plate with Si–C and O–C bonds. Because the surface of quartz glass polishing plate was always irradiated by plasma during PAP, it is assumed that the O-removed Si atoms on the quartz glass surface can be rapidly reoxidized by O atoms generated by Ar-based oxygen plasma, thus achieving a high MRR of SCD. More specific and accurate material removal mechanism in PAP will be investigated in our future research.

In this study, PAP using a quartz glass polishing plate was applied to polish a 20-mm square large mosaic SCD (100) substrate prepared by microwave plasma CVD. A very high MRR of 13.3 μm/h and a flatness of less than 0.5 μm were obtained. No surface structure dependent on crystal orientation was observed on the surface of the substrate after the PAP process, and a surface roughness of 0.4 nm *S*q was confirmed by AFM measurements. The results of Raman spectroscopy showed that there were no residual stress and non-diamond components. The abovementioned results show that the polishing of a large sized mosaic SCD substrate by PAP is very promising as a highly efficient and damage-free polishing technique.

## Methods

### Material

The mosaic SCD (100) substrate used in this experiment was prepared by the following procedures. At first, four 10-mm square clone substrates were produced from an identical 10-mm square single seed substrate. Then, the four clone substrates were arrayed with a gap of less than 500 μm to produce the clone mosaic substrate by additional CVD growth. Finally, a lift-off process was applied to obtain a free-standing clone mosaic SCD substrate^2^. The exact size of the mosaic SCD (100) substrate used in this study was 21 mm × 19 mm × 1 mm. Because the clone mosaic SCD substrate is made by joining small pieces, it is easy to break at the joint part when a mechanical polishing method, such as scaife, that applies excessive stress is applied. Therefore, the application of a technique with a low polishing pressure but a high polishing efficiency is required for polishing a clone mosaic SCD substrate.

### PAP experimental setup

Details of the PAP apparatus are described in reference^8^. The PAP apparatus is composed of plasma generation and mechanical removal parts. Both parts are installed in a vacuum chamber where the gas pressure can be controlled. The plasma generation part is composed of an upper electrode made of an aluminum alloy and a lower rotary table made of an aluminum alloy. The quartz glass polishing plate was installed on the lower rotary table. An Ar-based oxygen plasma was generated by applying an electric field of radio frequency (*f* = 13.56 MHz) to the upper and lower electrodes, and the electric power, gas pressure, argon flow rate, and oxygen flow rate were 100 W, 0.8 kPa, 200 sccm, and 30 sccm, respectively. In the mechanical removal part, the mosaic SCD substrate was fixed on the upper rotary table and pressed against the polishing plate. During PAP, the polishing plate irradiated by plasma and the SCD substrate were respectively driven by a speed-controlled motor to rotate in opposite directions with respect to each other. The relative sliding speed between the polishing plate and the SCD substrate was 1.2 m/s, and the rotation speed of the SCD substrate holder was 20 rpm.

### Surface characterization

The polishing characteristics of mosaic SCD substrate by PAP were evaluated by laser interferometer (GPI XP/D, Zygo), scanning white light interferometer (SWLI, NewView 8300, Zygo), atomic force microscope (AFM, SPM 9700, Shimadzu), and confocal Raman microscopy (RAMANtouch, Nanophoton) from the viewpoints of flatness, surface roughness, and crystallinity.
